# Adipokines and Adipose Tissue-Related Metabolites, Nuts and Cardiovascular Disease

**DOI:** 10.3390/metabo10010032

**Published:** 2020-01-11

**Authors:** Camila Weschenfelder, Alexandre Schaan de Quadros, Julia Lorenzon dos Santos, Silvia Bueno Garofallo, Aline Marcadenti

**Affiliations:** 1 Graduate Program in Health Sciences (Cardiology), Institute of Cardiology of Rio Grande do Sul/University Foundation of Cardiology (IC/FUC), Princesa Isabel Avenue, 395, Porto Alegre, Rio Grande do Sul 90040-371, Brazil; camilawesche@gmail.com (C.W.); ppgfuc@cardiologia.org.br (A.S.d.Q.); julia.lorenzon@gmail.com (J.L.d.S.); garofallo.silvia@gmail.com (S.B.G.); 2 HCor Research Institute, Coracao Hospital (IP-HCor), Abílio Soares Street, 250, São Paulo, São Paulo 04004-05, Brazil

**Keywords:** adipose tissue, adipokines, nuts, cardiovascular diseases, acetate, propionate, uric acid

## Abstract

Adipose tissue is a complex structure responsible for fat storage and releasing polypeptides (adipokines) and metabolites, with systemic actions including body weight balance, appetite regulation, glucose homeostasis, and blood pressure control. Signals sent from different tissues are generated and integrated in adipose tissue; thus, there is a close connection between this endocrine organ and different organs and systems such as the gut and the cardiovascular system. It is known that functional foods, especially different nuts, may be related to a net of molecular mechanisms contributing to cardiometabolic health. Despite being energy-dense foods, nut consumption has been associated with no weight gain, weight loss, and lower risk of becoming overweight or obese. Several studies have reported beneficial effects after nut consumption on glucose control, appetite suppression, metabolites related to adipose tissue and gut microbiota, and on adipokines due to their fatty acid profile, vegetable proteins, l-arginine, dietary fibers, vitamins, minerals, and phytosterols. The aim of this review is to briefly describe possible mechanisms implicated in weight homeostasis related to different nuts, as well as studies that have evaluated the effects of nut consumption on adipokines and metabolites related to adipose tissue and gut microbiota in animal models, healthy individuals, and primary and secondary cardiovascular prevention.

## 1. Introduction

Obesity is an established risk factor that predisposes to metabolic abnormalities and cardiovascular disease (CVD) [1,2,3]. Worldwide, a continuous increase in obesity incidence [4] and the cardiometabolic consequences [3], together with the knowledge about adipose tissue dysfunction (due to fat accumulation) on CVD [5], stimulate interest regarding the study of possible associated mechanisms.

White adipose tissue (WAT) plays a central role in controlling body energy balance, glucose homeostasis, insulin signaling [6], and produces a significant number of polypeptides called adipokines. Since the discovery of its most characteristic secretory product, leptin [7,8], WAT has been considered by many authors to be an endocrine organ [6], and a number of other signaling mediators secreted by adipose tissue have been identified since then. However, in addition to adipokines, other factors released by WAT, such as metabolites, lipids, non-coding RNAs, and extracellular vesicles, participate in the process of maintaining systemic homeostasis through communication between adipose tissue and other organs such as the intestine [9] and vascular system [10].

Interactions between intestinal microbiota, development of obesity, and associated diseases such as CVD have been evidenced [11,12]. Communication between the intestine and other organs—including adipose tissue and the vascular system—occurs, among other forms, through metabolite formation by the microbiota. These include short chain fatty acids (SCFA) and trimethylamine (TMA) [13], which may contribute to cardiometabolic disease pathophysiology [14]. In this sense, one of the main changes in the intestinal microbiota of individuals with metabolic syndrome is the reduced ability to produce SCFA from carbohydrates, which, in turn, is related to metabolic dysfunction of the host organism but not to obesity itself [15]. However, food-induced intestinal microbiota modifications and some specific nutrients appear to benefit the host [15,16].

Nuts are foods that contain a high energy density due to their nutritional composition, which is characterized by a high percentage of unsaturated fatty acids. Despite the high energy density, they are not associated with increased body weight, but with several benefits on cardiovascular health [17,18,19]. In addition to their high content of polyunsaturated fatty acids (PUFA) and monounsaturated fatty acids (MUFA), phytochemicals, dietary fibers, magnesium, L-arginine, and antioxidants compose these foods. Together, these nutrients act by modulating the intestinal microbiota [20,21] and are precursors of a series of metabolites [22,23]. In addition, nut consumption is inversely associated with the incidence of CVD, coronary artery disease (CAD), atrial fibrillation, as also with CVD mortality, CAD mortality, and stroke mortality [24].

Our purpose is to briefly review the possible interactions between the intake of different nuts, adipokines, and metabolites associated with WAT in the context of CVD and their risk factors. In addition, we present clinical studies in humans that have evaluated the effect of nut consumption on adipokines, adipose tissue-related metabolites, and intestinal microbiota in healthy individuals, and on primary and secondary cardiovascular prevention.

## 2. Nut Composition and Its Implication on Body Weight and Cardiometabolic Health

Nuts are thick, dried fruits [25] such as walnuts, almonds, pecans, Brazil nuts, cashews, pistachios, hazelnuts, and macadamia nuts [26]. Peanuts and baru almonds, although similar to nuts, are foods classified botanically as pulses [25]; however, they have as many benefits as much as true nuts.

Nutritionally, nuts have high concentrations of fats (40% to 60% of unsaturated fatty acids [UFA]) and protein (8% to 20%), presenting a good aminogram, except for lysine, methionine, and cysteine [25]. Regardless of the nut type, lipids are mainly composed of MUFA and PUFA (>75% of the total lipids) and analysis of fatty acid composition indicates that oleic acid (C18:1) is the main constituent of MUFA, and linoleic acid (C18:2) is the major PUFA [26]. PUFA and MUFA content of nuts play a role in glucose control and appetite suppression [27,28,29,30,31,32,33], in reducing plasma lipids [34,35,36,37,38], in inflammatory processes and their resolution [39,40,41,42], and in antioxidant defense against reactive oxygen species [43].

In addition, they deliver good levels of phytosterols, alpha-tocopherol, antioxidants, phenolic compounds, and dietary fibers [25,44,45], nutrients that in synergy seem to contribute to cardiovascular health [46]. Dried nuts also contain microRNAs (miR156c and miR159a) that exert an anti-inflammatory action by targeting TNF-α receptor in mammalian adipose tissue. In mice, these molecules were associated with a reduction of inflammatory cytokines in adipocytes and visceral adipose depots upon different pro-inflammatory conditions such as hypoxia, cellular hypertrophy, diet-induced obesity, in association with the downregulation of the TNF-α inflammatory signaling pathway [47].

Table 1 presents the amounts of energy, fats, dietary fiber, selenium, and phenolic compounds in 1 portion (30 g) of the main nuts.

The unique composition of nuts seems to explain the beneficial cardiometabolic effects observed in diets supplemented with this food [17,18,19]. In this sense, a series of clinical studies and systematic reviews have already shown that daily nut intake reduces body weight or does not incur increased adiposity [52,53,54,55,56,57,58,59], despite controversial results. For instance, supplementation of 40 g/day of hazelnut in 24 healthy subjects [54] did not decrease participant’s body weight (*p* = 0.46), but in overweight individuals, body weight and body mass index (BMI) were reduced after ingestion of 42.5 g/day of mixed nuts (almond, cashews, hazelnut, pecan, Brazil nuts, macadamia nuts, pistachios, walnut, and peanuts) for 8 weeks [55]. In subjects at high cardiovascular risk (such as diagnosis of type 2 diabetes mellitus [T2DM]), supplementation of 56 g/day of almonds compared to an isocaloric carbohydrate cookie for 8 weeks did not change anthropometric, body composition, or liver fat volume variables [60]. Similarly, cashew nut supplementation (10% of diet energy) for 8 weeks among 50 diabetic patients did not change participants’ body weight, BMI and waist circumference compared to the control diet (no supplementation) [61]. In individuals with established CVD, supplementation of 10 g/day of almonds for 12 weeks (10 g/day) also did not decrease adiposity [62].

Daily and morning intake of 44 g of pistachio for 12 weeks by healthy premenopausal women (*n* = 60) did not result in increased body weight and BMI [53]. However, pistachio consumption resulted in a compensatory reduction in energy intake, representing 26.3% of the extra energy provided by supplementation. A similar effect was observed in 137 individuals at increased risk for T2DM, who consumed 43 g of almonds daily for 4 weeks [63]. It was observed that almond supplementation significantly reduced hunger and desire to eat while eating intermediate meals, and there was no weight gain.

Despite not completely understanding the possible mechanisms that explain the relationship between nut consumption and lower adiposity, it is suggested that beyond appetite control (by regulating gut hormones [50,64,65]) and displacement of unfavorable nutrients (nut supplementation studies have shown improved dietary quality, particularly when nuts are consumed as a snack [50,66,67]), nuts may benefit weight-loss interventions and protect against weight gain by (1) increasing diet-induced thermogenesis (due to high UFA and protein content [50,68]); (2) lower availability of metabolizable energy (walnuts, pistachios and almonds seem to have an overestimated caloric content and lower metabolizable energy value than previous reported [50,69,70,71]); (3) antiobesity action of bioactive compounds [50,72]; and (4) improving functionality of the gut microbiome through maintaining integrity of the enteric barrier, improving anti-inflammatory status, and increasing butyrate synthesis [50] due to nuts’ prebiotic function [73,74].

Changes in gut microbiota composition (to an unfavorable gut microbial environment), or gut dysbiosis, have been linked to CVD, obesity, and T2DM [11,75]. In this sense, metabolites produced by the microbiota in obese individuals can affect cardiovascular health by enhanced inflammatory response [76,77], insulin resistance [78,79], liver fat accumulation [80], and increased plasma lipid levels [81,82].

On the other hand, adipose tissue produces and secretes several adipokines that play a role in energy homeostasis, carbohydrate and lipid metabolism, control of thermogenesis, reproduction, and immunity, and influence cardiovascular function [83,84]. Besides adipokines, WAT produces and secretes metabolites with systemic effects such as in glucose metabolism [85,86], basal metabolic rate [87], oxidative stress [88], vasodilation [89] and inflammation [90], and that may contribute to the pathophysiology of obesity and CVD.

### 2.1. Adipose Tissue-Related Metabolites, Cardiovascular Risk, and Nuts

#### 2.1.1. Uric Acid

Uric acid, a product derived from purine degradation, is produced by adipose tissue, liver, and skeletal muscle and is mainly excreted by the kidneys and liver [91]. In adipose tissue, uric acid is produced through xanthine oxidoreductase (XOR); in animal models, production is increased in obesity [92] possibly due to hypoxia of hypertrophied adipose tissue and, consequently, increased XOR activity. Its elevated level has been related to the higher risk for prehypertension [93], high blood pressure, dyslipidemia, and impaired glucose metabolism [85], which are strongly related to CVD. It has been identified that high concentrations of uric acid are related to an increased risk for CAD, heart failure (HF) and atrial fibrillation. In addition, hyperuricemia has already been associated with higher CVD mortality [94,95].

Among mechanisms that explain the deleterious effect of elevated uric acid levels on cardiovascular health are increased oxidative stress, reduction of available nitric oxide and consequent endothelial dysfunction, promotion of local and systemic inflammation, vasoconstriction and smooth muscle cell proliferation, insulin resistance, and metabolic dysregulation [88].

An acute protocol [96] investigated the postprandial effect of eating a walnut-based meal (consisting of 90 g of shelled walnut and 250 mL of distilled water) compared to a meal consisting of olive oil, white bread, egg white powder, and 250 mL distilled water—both adjusted for calories for each participant. No difference in plasma uric acid concentrations was observed between interventions in healthy subjects. In a randomized controlled trial, supplementation of 10 g/day of American almonds or 10 g/day of Pakistani almonds, both consumed prior to breakfast, reduced serum uric acid by 18% and 14% in patients with CAD after 12 weeks, respectively (*p* < 0.05), compared to the control group, which did not eat almonds [97].

#### 2.1.2. Uridine

Uridine is the nucleoside of the uracil pyrimidine base and is produced by adipose tissue and the liver, where it is also degraded [98,99]. While the liver produces uridine in the fed state, adipose tissue produces it in the fasting state. Plasma uridine concentrations are elevated during fasting and fall rapidly in the postprandial state as nutrient ingestion triggers the release of bile. Fasting, coupled with increased plasma uridine, causes a hypothalamic response that culminates in body temperature reduction, while bile-mediated uridine release promotes a decline in plasma uridine and improves insulin sensitivity [87].

In an experimental model [87], uridine administration increased plasma leptin levels, decreased basal metabolic rate, improved glucose tolerance in older rats on high-fat diets, and high doses resulted in reduced body temperature, a mechanism that seems to involve leptin signaling.

Disturbances of uridine homeostasis, both up and down, appear to be deleterious [9]. Prolonged (16 weeks) dietary supplementation of uridine in rats promoted liver fat accumulation and glucose intolerance [86]. Supplementation for five days altered hepatic protein glycosylation [100] and promoted liver fat accumulation [101]. In contrast, mice with overexpression of the protein X-box binding protein 1, which is a transcription factor for de novo uridine synthesis, exhibited high levels of circulating and adipose tissue uridine, higher energy expenditure, lower body weight, lower temperature, and protection against obesity even when on a high-fat diet or in a model of decreased leptin expression (*ob* gene knockout) [99].

However, there is still little knowledge about the effects of short- and long-term uridine homeostasis disorders on systemic metabolism, as well as their use as a therapeutic resource [9].

#### 2.1.3. Palmitic Acid Methyl Ester (PAME)

PAME is a hydrophobic, low molecular weight fatty acid metabolite secreted by adipose tissue [102] that is capable of inducing vasodilation via potassium channel activation [89]. It also appears to have anti-inflammatory and antifibrotic effects by inhibiting nuclear factor kappa B (NF-kB) [90].

Methyl palmitate administration in rats undergoing anticancer treatment demonstrated cardioprotection from the effects of treatment cardiotoxicity, a fact attributed to methyl palmitate’s ability to suppress oxidative stress and disrupt the toll-like receptor-4 (TLR4)/NF-kB pathway with a consequent reduction in apoptosis [103].

PAME seems to play an important role between peripheral adipose tissue and vasculature. The anti-contractile function of peripheral adipose tissue is reduced in spontaneously hypertensive rats, as is PAME release. Both mechanisms seem to contribute to hypertension genesis [102].

The effects of nut consumption on uridine and PAME levels are unknown.

### 2.2. Adipokines, Cardiovascular Risk, and Nuts

#### 2.2.1. Leptin

Leptin is a product of mature adipocytes [7,8], acting mainly in the brain [104]. Leptin levels are reduced in fasting periods, triggering different mechanisms such as increased appetite through stimulation of neuronal hypothalamic pathways [105], decreased thyroid hormone production [106], inhibition of the reproductive axis [107], and depression of the immune system [108]. At high concentrations, leptin stimulates oxidative stress, inflammation, thrombosis, angiogenesis, and atherogenesis, which predispose CVD [109]. In contrast, voluntary physical activity reduces leptin signaling to the stromal hematopoietic bone marrow niche, consequently decreasing chronic hematopoietic output of inflammatory leukocytes and protecting from CVD [110].

Results are conflicting regarding leptin concentrations and incidence of CVD. In a cohort of 1905 subjects and a 7.6-year follow-up, a standard deviation of increased leptin levels was not correlated with CVD incidence (hazard ratio (HR) = 0.87; 95% CI = 0.68–1.11; *p* = 0.26) [111]. In another cohort of patients with coronary artery disease, increased leptin concentration was a predictor of cardiovascular mortality and nonfatal acute myocardial infarction (MI) in women (HR = 1.28; 95% CI = 1.01–1.62; *p* = 0.04), but not in men [112].

In a meta-analysis that included 13 cohort and case-control studies, totaling 4257 CVD patients and 26,710 non-CVD controls, high leptin levels were not independently associated with CAD in women (odds ratio (OR) = 1.03; 95% CI = 0.86–1.23) and men (OR = 1.09; 95% CI = 0.95–1.26) or with stroke in women (OR = 1.13; 95% CI = 0.87–1.47) and men (OR = 0.80; 95% CI = 0.59–1.09) [113].

Regarding nut consumption and leptin levels, results are also conflicting. For instance, mixed nuts supplementation decreases leptin concentrations in overweight individuals [64], but not a walnut-rich meal in healthy individuals [114] or a 48 g walnut smoothie in patients with obesity [65]. The effect of nut intake on leptin concentrations was summarized in a systematic review [115], in which consumption of different nut doses (studies ranged from 0.5 to 128 g/day) was associated with reduced leptin levels (−0.71 mg/dL; 95% CI = −1.11 to −0.30).

#### 2.2.2. Adiponectin

Adiponectin is a hormone associated with benefits on cardiometabolism, exerting anti-inflammatory, antioxidant, anti-atherogenic, pro-angiogenic, and vasoprotective effects [116]. Adiponectin increases insulin sensitivity [117], an effect also observed after weight loss and the consequent increase in plasma levels [118]. Simvastatin treatment, often observed in patients with CVD, increases adiponectin levels over 12 weeks but not below 8 weeks [119].

Meta-analysis of 17,598 adults evaluated the association between adiponectin levels and the risk of developing high blood pressure [120]. Each 1 µg/mL increase in adiponectin levels was associated with a 6% reduction in the risk of hypertension (OR = 0.94; 95% CI = 0.91–0.96; *p* < 0.001). Adiponectin levels have already been associated with CAD risk through meta-analysis of case-control and cohort studies. In this study, with 14,960 individuals and an incidence of 4132 cases of CAD, an inverse relationship was observed between high adiponectin levels and the incidence of CAD (HR = 0.83; 95% CI = 0.69–0.98; *p* = 0.031) [121]. However, in other studies, higher adiponectin concentrations were not associated with other outcomes such as carotid plaques, ischemic stroke, and mortality [122].

High adiponectin levels do not necessarily improve outcomes in CVD patients [123]. Meta-analysis [124] conducted among 862 HF patients noted that increased adiponectin levels were associated with higher all-cause mortality (RR = 2.05; 95% CI = 1.22–3.43) and increased combined outcomes of readmission and death (RR = 2.22; 95% CI = 1.38–3.57). In these patients, increased adiponectin may be consistent with wasting observed in cardiac cachexia, which is associated with worse outcomes in HF [125].

Improvement in adiponectin levels with pistachio supplementation (20% of diet energy) has been observed in humans with metabolic syndrome [126], as well as a walnut-rich meal in healthy individuals [114]. However, supplementation of 20 g baru almonds for 8 weeks among obese and overweight individuals did not improve adiponectin levels [127], nor did mixed nuts supplementation [64] or a 48 g walnuts smoothie in patients with obesity [65]. A systematic review concluded that different nut doses (studies ranged from 0.5 to 128 g/day) do not increase levels of adiponectin (−0.60 mg/dL; 95% CI = −1.88 to 0.68) [115].

#### 2.2.3. Resistin

In humans, resistin is produced by adipose tissue [128] and expressed by peripheral blood mononuclear cells and macrophages [129] under inflammatory stimulation by lipopolysaccharide (LPS), tumor necrosis factor alpha (TNF-α), interleukin (IL)-6, IL-1β, and resistin itself [128]. Resistin secretion triggers an inflammatory response by releasing proinflammatory cytokines [130]. It participates in the atherosclerotic process, promoting proliferation and migration of endothelial cells and smooth muscle vascular cells, increasing endothelial permeability and consequently monocyte adhesion and infiltration [131]. Resistin levels increase in the presence of obesity and appear to be causally related to T2DM development [132].

Through meta-analysis [133] consisting of 718 hypertensive and 645 normotensive individuals, a positive association was observed between resistin concentrations and hypertension. The association was stronger among diabetic patients when compared to a non-diabetic population. Another meta-analysis observed an association between resistin levels and CAD [134]. Among 2070 subjects, compared to disease-free controls, higher resistin levels were found among subjects with stable angina (standardized mean difference (SMD) = 1.97; 95% CI = 1.15–2.79), unstable angina (SMD = 2.54; 95% CI = 1.76–3.31) and MI (SMD = 3.62; 95% CI = 2.62–4.62).

Resistin levels have already been associated with higher mortality. Among 7 studies evaluating total mortality (*n* = 4016 and 961 events) and 6 studies evaluating CVD mortality (*n* = 4187 and 412 events), the increase of 1 standard deviation in resistin concentration increased the risk of total mortality (HR = 1.28; 95% CI = 1.07–1.54; *p* = 0.008) and CVD mortality (HR = 1.32; 95% CI = 1.06–1.64; *p* = 0.013) [135].

No differences in resistin levels were observed in healthy young adults when submitted to a walnut-rich meal, a butter-enriched meal, or an olive-oil-enriched meal [114].

#### 2.2.4. Progranulin

Progranulin is a protein related to neurodegenerative and metabolic diseases [136]. In peripheral tissues, excess progranulin is associated with obesity and insulin resistance [137,138]. Considering its potential effect on the cardiovascular system, progranulin has been associated with angiogenesis, cell proliferation, and inflammation [139].

However, progranulin has already been associated with vascular endothelium protection in cell culture, where it inhibited LPS-mediated inflammation in endothelial cells [140]. In rats, progranulin suppression led to the development of more severe atherosclerotic lesions when compared to non-progranulin suppressed animals, an effect attributed to increased expression of inflammatory cytokines, adhesion molecules, reduced expression of endothelial nitric oxide synthase, and cholesterol accumulation in macrophages [141].

In a cohort study of 1046 subjects, serum triglycerides levels were positively correlated with progranulin concentrations (β = 0.069; *p* = 0.037) [142]. Among 216 individuals of another cohort study, with recent ischemic stroke and 100 controls, progranulin was able to predict mortality independent of other factors [143].

Among 362 adults with acute coronary syndrome (ACS) (*n* = 69), stable angina (*n* = 85) and control subjects (*n* = 208), progranulin concentrations did not differ between groups but were negatively correlated with HDL-c (r = −0.105, *p* = 0.048) [144]. Among individuals with (*n* = 44) and without (*n* = 83) metabolic syndrome, progranulin levels were associated with higher concentrations of C-reactive protein (CRP), IL-6, number of metabolic syndrome components, and increased intima*-*media thickness in individuals without metabolic syndrome [145].

#### 2.2.5. Omentin-1

Omentin-1 is expressed in visceral adipose tissue (VAT) cells [146] and is negatively associated with intima-media thickness, waist circumference, body mass index (BMI), systolic blood pressure (SBP), fasting glucose, and homeostatic model assessment of insulin resistance (HOMA-IR). Low concentrations of omentin-1 contribute to insulin resistance pathogenesis, T2DM, and CVD in overweight patients [147].

Among 193 postmenopausal women, lower levels of omentin-1 were identified among women with CAD (*n* = 110) when compared to women without CAD (*n* = 83) (247.5 ± 127.4 vs. 506 ± 246 ng/mL) and reduced omentin-1 levels were an independent risk factor for disease severity as measured by the SYNTAX score [148]. Among 225 patients with severe carotid stenosis and a low degree of stenosis, omentin-1 was not associated with plaque vulnerability after adjustment in multivariate analysis [149].

One study evaluated healthy and obese subjects with T2DM and coronary stenosis and found that they had lower omentin-1 levels when compared to healthy subjects (0.19 ± 0.05 vs. 0.54 ± 0.12 ng/mL; *p* < 0.05) [150]. Omentin-1 levels were also negatively correlated with BMI, glycated hemoglobin (HbA1c), total cholesterol, TAG, LDL-cholesterol and VLDL-cholesterol, and positively with HDL-cholesterol in this sample.

However, in 2084 participants from a cohort-nested case-control study with 50 prevalent CVD cases and 350 incident cases with a median follow-up of 8.2 years, omentin-1 was not associated with risk for MI (HR per doubling omentin-1 = 1.17; 95% CI = 0.79–1.72; *p* = 0.43), but with a higher risk for stroke (HR per doubling omentin-1 = 2.22; 95% CI = 1.52–3.22; *p* < 0.0001) [151].

The effects of nut consumption on progranulin and omentin-1 levels are unknown. Table 2 summarizes the main human clinical studies that evaluated the effect of different nuts on indicators of adiposity, adipokines, and other parameters related to body weight homeostasis.

## 3. Metabolites Formed by Microbiota, Adiposity, Cardiovascular Risk, and Nuts

Pathological conditions, such as obesity, can significantly reduce or increase communication between different organs. In this sense, a number of mechanisms seem to explain the relationship between adipose tissue and intestine through interaction with the intestinal microbiota [16]. Among them are included the formation of metabolites, such as SCFA (acetate, propionate and butyrate) [154], and the production of intermediate metabolites, such as lactate and TMA.

Adiposity indexes and WAT compartments have been associated with urinary metabolites involved in gut microbiota metabolism [155], such as choline (its metabolism by the gut microbiota results in the production of TMA, which upon absorption by the host is converted in the liver to trimethylamine-N-oxide [TMAO]) [156,157], ethanolamine (its utilization by certain gut bacteria affects lipid metabolism and SCFA biosynthesis [158], dimethylamine [159] and glutamine [160].

On the other hand, in a dietary intervention trial, circulating choline decreased among participants who had greater improvements of adiposity after eating a low-calorie weight loss diet and more significant decreases in choline were strongly associated with larger reductions in body fat composition, fat distribution, and energy expenditure [161]. It has been also shown that gut microbiota controls the expression of the miR-181 family in white adipocytes during homeostasis to regulate key pathways controlling adiposity, insulin sensitivity, and WAT inflammation in mice [162].

### 3.1. Acetate

Acetate is a product of the liquid fermentation of most anaerobic intestinal bacteria, and it is also produced by acetogenesis, which has the highest concentration of SCFA in the intestinal lumen [163]. An in vitro study [164] evaluated the effect of acetate on human WAT-derived stem cells and found that acetate had an antilipolytic effect, which was achieved by reducing hormone-sensitive lipase phosphorylation.

In rats [165], acetate stimulated a number of mechanisms in different peripheral tissues. In the liver, it reduced fat deposition by reducing circulating free fatty acids, reduced de novo lipogenesis, and increased mitochondrial efficiency, while in adipose tissue, it induced browning leading to a reduction in body adiposity [165].

The effects of acetate in animal models demonstrate that it has a beneficial potential on metabolism via secretion of hormones such as glucagon like peptide-1 (GLP1) and peptide YY (PYY), affecting appetite, reduction of lipolysis and secretion of proinflammatory cytokines, and increasing energy expenditure and fat oxidation [166].

Composition of intestinal microbiota is linked to adipose tissue browning and insulin action in morbidly obese individuals, possibly via circulating acetate [167]. In grade III obese, the firmicutes RA strain was negatively correlated with serum glycated hemoglobin (HbA1C) and serum triglycerides concentrations and was positively associated with brown adipocyte markers such as the PR domain containing 16 (PRDM16), uncoupling protein 1 (UCP1), and type II iodothyronine deiodinase in subcutaneous adipose tissue. This strain was positively associated with plasma acetate levels, which was related to PRDM16 mRNA in subcutaneous adipose tissue and insulin sensitivity [167].

### 3.2. Propionate

Carbohydrate fermentation results in propionate formation by intestinal bacteria in two ways: via succinate and via propanediol [163]. In addition, amino acid fermentation appears to contribute to propionate formation [163].

In animal models, propionate has been associated with improved lipid metabolism in rats given a high-fat diet [168,169]. In addition, propionate treatment significantly decreased body weight, fat mass, and inguinal WAT volume, suggesting that propionate could reverse fat-induced lipid accumulation [168]. Propionate also appears to play an important role in reducing hepatic triglycerides, improving insulin sensitivity, and increasing the formation of odd-chain fatty acids [170].

Oral administration of propionate in two animal models, one hypertensive and one atherosclerotic, demonstrated anti-inflammatory properties limiting CVD progression in both. Propionate influenced helper T-cell homeostasis, reducing cardiac hypertrophy and fibrosis, susceptibility to arrhythmias, and atherosclerotic lesions. At the same time, propionate exerted an antihypertensive effect in both animal models [171].

In overweight adults, propionate levels in the intestinal colon were associated with weight gain prevention by increasing hormone release such as PYY and GLP-1 and, therefore, reduced energy intake [172]. In addition, propionate supplementation improved insulin sensitivity and reduced proinflammatory cytokine IL-8 in overweight or obese adults [173].

### 3.3. Butyrate

Two different routes follow for butyrate formation: via butyryl-CoA:acetate-CoA transferase or via phosphotransbutyrylase and butyrate kinase [174]. Similar to propionate, amino acid fermentation also contributes to butyrate formation [163].

In rats on a high-fat diet, butyrate supplementation induced the activation of AMP-activated 5′Protein Kinase (AMPK) and glucose transporter 4 (GLUT4) in the adipose tissue, attenuated diet-induced dysbiosis, promoted biosynthesis of resolvin E1 and lipoxin (anti-inflammatory lipid mediators) [175], attenuated weight gain, adiposity, adipocyte hypertrophy, inflammation, and leptin secretion [176]. In the same animal model, butyrate supplementation appears to induce lipolysis in WAT mediated by activation of β3-adrenergic receptors [177] and regulates gene expression related to intestinal cholesterol absorption resulting in attenuation of atherosclerosis [178].

Chronic butyrate supplementation in rats prevented diet-induced obesity, hyperinsulinemia, hypertriglyceridemia, and hepatic steatosis, effects attributed to reduced dietary intake [179]. The reduction appears to be due to suppression of neuropeptide Y expression in the hypothalamus, resulting in changes in the gut–brain neural circuit. In addition, butyrate supplementation promoted fat oxidation and activated brown adipose tissue (BAT), effects that can be explained by increased sympathetic flow to this compartment [179].

In humans, butyrate supplementation does not appear to benefit individuals with metabolic syndrome but appears to have a beneficial effect on glucose metabolism in lean men, who showed improvement in peripheral and hepatic insulin sensitivity, suggesting different use and flow of SCFA during obesity and insulin resistance [180].

In a crossover clinical trial [181], the impact of daily consumption of 85 g of almonds or pistachio for 18 consecutive days was assessed, and higher fecal content of butyrate-producing bacteria was observed in both interventions—but stronger effects were observed after pistachio consumption. Healthy individuals undergoing an isocaloric diet intervention containing 42 g of walnuts daily for three weeks had a higher relative abundance (49–160%) of fecal microbiome, increasing the relative abundances of Firmicutes species in butyrate-producing Clostridium clusters XIVa and IV, including Faecalibacterium and Roseburia [182].

A study evaluating the prebiotic potential of whole almonds and defatted almonds using an in vitro gastrointestinal fermentation model showed a higher concentration of Eubacterium rectale after digestion of whole almonds compared with commercial prebiotics, and concomitant increase in butyrate concentration with both almonds [74].

### 3.4. Combined Propionate, Butyrate, and Acetate

Some studies have evaluated the use of the three major SCFA in combination or alone in the same protocol. A study in rats [183] demonstrated that isolated or mixed supplementation of propionate, butyrate, and acetate can modulate adiponectin and resistin gene expression in obesity via epigenetic regulation. Rats eating a high-fat diet had reductions in adiponectin and resistin mRNA levels in adipose tissue, which were reversed with supplementation of SCFA. In addition, changes in SCFA-induced adiponectin and resistin expression have been associated with changes in DNA methylation [183].

In pigs [184] supplemented with SCFA separately or mixed, oral administration of SCFA attenuated fat deposition via reduced lipogenesis and increased lipolysis in different tissues. Supplementation of SCFA reduced the concentration of TAG, total cholesterol, LDL-cholesterol, insulin, and liver total fat; increased serum leptin concentrations; reduced mRNA expression of fatty acid synthase and transcription factor binding to sterol regulatory element 1; and increased carnitine palmitoyl transferase I (CPT-1α) mRNA expression in liver and VAT.

Overall, these results suggest a potential protective effect of SCFA against obesity-associated cardiometabolic abnormalities. However, higher levels of SCFA in stools appear to be associated with lower intestinal microbiota diversity and poor cardiometabolic health, and higher systemic inflammation, blood glucose, dyslipidemia, obesity, hypertension and high excretion of SCFA in stool may be a marker of cardiometabolic dysregulation [185].

An increase in SCFA (in percentages) after fermentation of mixed nuts (hazelnuts, almonds, macadamia, pistachios, and walnuts) has been demonstrated with a modification of the acetate/propionate/butyrate molar ratio from 57:24:19 to 48:24:28 [73].

### 3.5. Lactate

Lactate can be metabolized to acetate, propionate, and butyrate by various organisms [163]. Many different intestinal bacteria such as lactobacilli, bifidobacteria, enterococci, and streptococci produce lactate [186]. Several tissues use lactate as an energy substrate, such as the heart [187], WAT and BAT [188]. Variations in its synthesis rate, blood transport, and final availability modulate important metabolic substrate changes [189]. Adipocytes contribute significantly to systemic lactate homeostasis, with important physiological and pathophysiological implications [190,191,192,193].

Cultivated adipocytes exposed to high glucose levels produce and secrete larger amounts of lactate; therefore, hyperglycemia appears to be related to higher lactate levels [191].

Higher circulating lactate levels in obese humans suggest a potential role of WAT in glycemic control [192]. The ability of WAT to produce lactate does not directly depend on its metabolic condition, but this production is a direct consequence of the activity of the lactate dehydrogenase (LDH) in tissue [189]. This activity is a direct correlate of the expression of major LDH-controlling genes, which appear to convert excess circulating glucose into 3C fragments as a means of controlling blood glucose and/or providing shorter chain substrates for use as energy sources in other tissues. [189].

Pistachio consumption (85 g/day) appears to decrease the number of lactic acid bacteria after 18 days when compared to the same quantity of almonds [181].

### 3.6. Trimethylamine N-Oxide (TMAO)

TMAO is a TMA derivative, produced in the gut by multiple nutritional substrates containing a TMA fraction such as choline, L-carnitine, γbutyrobetaine, and betaine. TMA produced in the gut is absorbed into circulation and converted in the liver by the enzyme flavin-containing monooxygenase 3, to TMAO [157].

TMAO is recognized as a risk factor for the incidence and progression of CVD [154,185,186] and has been related to a higher risk of cardiovascular events and all-cause mortality, regardless of traditional risk factors [194,195,196].

Adverse effects of TMAO on cardiovascular function have been associated with multiple mechanisms, including atherosclerosis promotion [197], reduction of reverse cholesterol transport, and defects in cholesterol metabolism in general [198,199]. In addition, it promotes endothelial dysfunction, exacerbates platelet reactivity, increases thrombosis, and affects the inflammatory response [200,201,202,203].

A 4-month randomized trial with prediabetic subjects evaluated the impact of a diet supplemented or not with 57 g pistachio and identified a significant reduction in urinary TMAO concentration (*p* = 0.034) after pistachio consumption [204]. In addition, pistachio supplementation reduced urinary concentration of dimethylamine (*p* = 0.044), a microbiota-derived metabolite formed from TMA [159] and predictor of mortality in individuals with and without a diagnosis of CAD [205].

**Figure 1:**
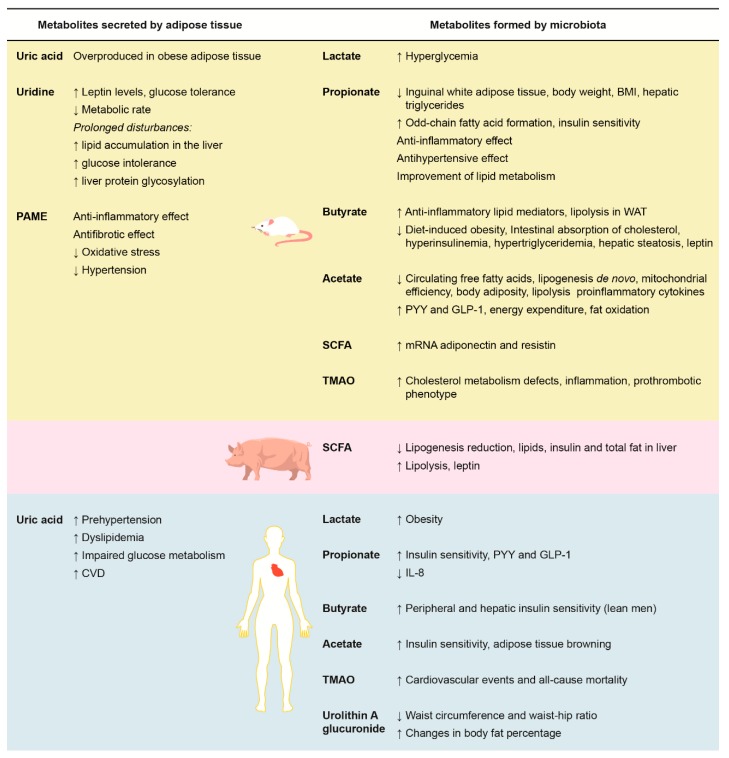
Effects of adipose tissue-secreted metabolites (**left**) and microbiota-formed metabolites (**right**) on rat, pig, and human metabolisms on adipose tissue and cardiovascular system parameters. BMI: body mass index; CVD: cardiovascular diseases; GLP-1: glucagon-like peptide-1; IL-8: interleukin 8; PAME: palmitic acid methyl ester; PYY: peptide YY; TMAO: trimethylamine N-oxide; WAT: white adipose tissue. ↑: increase; ↓: decrease.

Figure 1 summarizes the effects of adipose tissue secreted metabolites and intestinal microbiota metabolites on rat, pig, and human metabolisms, especially on parameters associated with adipose tissue and the cardiovascular system.

## 4. Nuts and Other Metabolites

Since nuts are sources of fats and a wide variety of micronutrients and phytochemicals, after ingestion, several of their constituents, as well as their derived metabolites, are found in the bloodstream and urine. These molecules appear to serve as markers of nut intake, where α-linolenic acid, urolithins, and 5-hydroxyindole-3-acetic acid appear to be markers of walnut intake, α-tocopherol and catechin-derived metabolites appear to be markers of almond intake, and selenium marks Brazil nut consumption [22].

Among healthy American men and women, 17 lipid metabolites were associated with 1 serving of nuts (28 g/day consumption) [23]. Positive associations were found for sphingomyelin, phosphatidylcholine, ceramides, and phosphatidylethanolamine, 3 of which were associated with peanut and peanut butter consumption (C24: 0 sphingomyelin, C24: 0 ceramide, and C22: 0 sphingomyelin). Negative associations were found with diacylglycerols, lysophosphatidylcholines, lysophosphatidylethanolamine, and cholesterol esters, consistent with favorable effects of nut consumption on lipid metabolism [23]. In obese individuals, consumption of 48 g/day of walnuts decreased harmful ceramide, hexosylceramide, and sphingomyelin concentrations related to cardiovascular risk [65].

Urolithin A glucuronide, a product derived from the biotransformation of walnut polyphenols in the gut, has been associated with lower severity of metabolic syndrome [206]. In a randomized clinical trial conducted among 50 individuals with metabolic syndrome who consumed mixed nuts (15 g walnuts, 7.5 g almonds, and 7.5 g hazelnuts) for 12 weeks, urolithin A glucuronide levels were inversely associated with waist circumference (r = −0.550, *p* = 0.005), waist-hip ratio (r = −0.409, *p* = 0.047), and positively associated with changes in body fat percentage (r = 0.456, *p* = 0.025).

In a crossover clinical trial [182] among 18 healthy subjects, after walnut supplementation for three weeks, serum campesterol was 10 μmol/mmol lower (6% reduction) during the walnut supplementation period compared to the control period (without supplementation), and lathoesterol concentration tended to decrease in the same period.

## 5. Nuts, Metabolites, and Adipose Tissue: Primary and Secondary Cardiovascular Prevention

With regard to different cardiovascular risk-related metabolites, a study by Würst et al. [207] used quantitative nuclear magnetic resonance imaging to identify biomarkers in CVD incidence, based on the metabolites evaluated in the National Finnish Study (FINRISK). Replication and improved risk prediction were assessed in the Southall and Brent Revisited (SABER) and the British Women’s Health and Heart Study. Among 68 lipids and metabolites evaluated, two of them were associated with increased risk for cardiovascular events after adjustment for other variables: elevated plasma phenylalanine and higher concentrations of MUFA. High levels of omega-6 fatty acids and docosahexanoic acid were associated with lower risk of CVD.

On the other hand, association of nut intake, metabolites, and cardiovascular prevention was especially evaluated in the Prevención con dieta Mediterranea (PREDIMED) study [208]. Firstly, changes in 202 basal lipid metabolites after one year of intervention with low-fat diet (control group) or Mediterranean diet (supplemented with extra virgin olive oil or mixed nuts) and their associations with cardiovascular events were evaluated among 230 cases of CVD patients and 790 controls without the disease. At the end of the follow-up period, a significant change in 20:3 cholesterol ester levels were observed only in the nut group. However, there was no significant difference regarding the risk of cardiovascular events between the groups, nor association with observed changes in metabolite levels [209].

Another case-control study [210] (231 CVD cases with 985 controls) derived from PREDIMED evaluated plasma levels of tryptophan, chirurenin, quinurenic acid, 3-hydroxyanthranilic acid, and quinolinic acid after one year of intervention and associations with cardiovascular events (nonfatal MI, nonfatal stroke, or cardiovascular death). Increased tryptophan levels after one year were associated with a lower risk of events (HR = 0.79; 95% CI = 0.63–0.98), and basal quinurenic acid concentration was associated with increased risk of MI and CAD death, but not at risk of stroke.

Thirdly, the association between plasma ceramides and CVD risk was assessed after 4.8 years of follow-up [211]. Extreme quartiles of plasma concentrations of ceramides C16:0, C22:0, C24:0 were compared, and a score was calculated by summing the concentrations. The highest quartile was associated with a 2.18-fold increased risk for combined cardiovascular events in the PREDIMED study. However, changes in ceramide concentration were not different between groups (low-fat diet or Mediterranean diet).

As discussed earlier, it has been already identified: (1) some metabolites correlated with nut consumption [23]; (2) changes in plasma metabolite profile directly correlated with adipose tissue and with cardiometabolic risk in humans [212]; (3) metabolites directly associated with risk for CVD [207]; and (4) nut-associated metabolites are effective in primary cardiovascular prevention, in a context of intervention with a Mediterranean diet [209,210,211]. These latest studies suggest that nuts may act beneficially on cardiovascular health by reducing cholesterol ester levels and increasing tryptophan levels. However, studies that specifically correlate the consumption of nuts, metabolites, and adipose tissue in secondary cardiovascular prevention are scarce, and this is an important field of research to explore further.

## 6. Conclusions

Metabolites, which are secreted by adipose tissue, formed by the intestinal microbiota or originated from nut components, appear to be related to adipokines, cardiometabolism, and CVD. Nut supplementation is associated with favorable outcomes in the cardiovascular system, without increasing adiposity. However, these results appear to be dependent on nut composition, dose, and duration of intervention. Due to their complex nutritional composition, several mechanisms seem to explain the possible benefits associated with nut consumption on adipose tissue and intestinal microbiota modulation, but their specific effects on the complex adipose tissue–gut microbiota-cardiovascular system network are not yet fully established. Interactions between nuts, adipose tissue, and adipokines/derived metabolites on cardiovascular health need further investigation, especially in the context of primary and secondary cardiovascular prevention through large randomized controlled trials.

## Figures and Tables

**Table 1:** Nutritional composition of different nuts in 30 g serving.

	Energy (kcal)	Total Fat (g)	MUFA (g)	Omega-3 (g)	Fiber (g)	Alpha-Tocopherol (mg)	Selenium (mg)	Phenolic Compounds (mg)
Peanut [48]	163.2	13.17	5.16	0.012	2.4	-	-	-
Walnut [49,50,51]	196.69	19.59	2.68	2.04	1.98	0.36	-	468.53–488.63
Almonds [49,50]	174.05	15	9.48	-	3.75	4.74	15.6	14.04–125.73
Pecan [48,49,50]	186	17.82	2.61	2.64	2.16	3.84	-	385.2–606.22
Brazil [48,49,50]	192.9	19.05	8.22	0.012	2.37	24.87	61.2	33.67–93.25
Cashew [49,50]	166.27	13.23	7.14	-	0.99	1.08	-	41.17–82.42
Pistachio [49,50]	168.39	13.65	7.05	-	3.18	4.36	25.5	260.73–498.25
Hazelnut [49,50]	188.91	18.25	13.72	-	2.9	9.44	27	87.52–251.10
Macadamia [50]	215.8	22.78	17.68	-	2.61	-	-	13.86–46.91
Baru almond [49]	155.41	12.31	15.32	0.66	4.17	-	-	-

MUFA: monounsaturated fatty acids.

**Table 2:** Clinical trials that evaluated the effect of different nuts on indexes of adiposity, adipokines, and other parameters related to body weight homeostasis.

Reference	Population	Sample Size	Design	Duration	Intervention Group	Control Group	Outcomes
Abbaspour, 2019 [55]	BMI of ≥27 kg/m^2^	54	RCT, parallel-arm	8 weeks	42.5 g/day mixed nuts	Isocaloric pretzel	↓ Body weight ↓ BMI → Waist circumference → Hip circumference → Waist-to-hip ratio
Di Renzo, 2019 [54]	Healthy volunteers	24	Clinical trial—pilot	6 weeks	40 g/day hazelnuts	Baseline	↓ Abdominal circumference → Body weight
Fantino, 2019 [53]	BMI 19–29.9 kg/m^2^ Pre-menopausal women	60	RCT, parallel-arm	12 weeks	44 g/day pistachio snack in the morning	Instructed not to consume pistachios	→ Body weight → BMI ↑ Satiety
Tan, 2013 [63]	Increased risk for T2DM -BMI >27 kg/m^2^ -Normal weight with a strong family history for T2DM	137	RCT, parallel-arm	4 weeks	43 g/day almonds	Avoid all nuts and seeds	→ Body weight ↓ Hunger ↓ Desire to eat
Bowen, 2019 [60]	Overweight and obese adults with elevated fasting blood glucose	76	RCT, parallel-arm	8 weeks	56 g/day almonds	Higher carbohydrate biscuit snack isocaloric	→ Body weight → BIA weight → BIA FFM → BIA body fat mass → BIA muscle mass → Waist circumference → SCAT → VAT → Liver Fat
Godwin, 2019 [64]	Healthy, obese and overweight adults	54	RCT, parallel-arm	Baseline and 20, 40, 60, 90, 120 min after snack consumption	42 g/day mixed nuts	Unsalted pretzels	↓ Leptin ↓ Ghrelin → Adiponectin → Cholecystokinin → PYY
Gulati, 2014 [126]	Metabolic syndrome	60	RCT, parallel-arm	24 weeks	20% of daily energy in pistachios	Control diet	→ Body weight ↓ Waist circumference ↑ Adiponectin
de Souza, 2019 [127]	Overweight and obese women	46	RCT, parallel-arm placebo-controlled trial	8 weeks	20 g/day baru almonds	800 mg/day of maltodextrin dispensed in sachet	→ Adiponectin
Damavandi, 2019 [61]	T2DM	50	RCT, parallel-arm	8 weeks	10% of daily energy in cashews	Control diet	→ Body weight → Waist circumference → IMC
Jamshed, 2015 [62]	CAD patients with optimal LDL cholesterol (≤100 mg/dL) and low HDL cholesterol (men ≤40 mg/dL and women ≤50 mg/dL)	150	RCT, parallel-arm	12 weeks	10 g/day Pakistani almonds 10 g/day American almonds *Both before breakfast*	No intervention	→ Body weight
Tuccinardi, 2019 [65]	Obese adults (BMI ≥ 30 kg/m^2^)	10	RCCT	5 days	48 g/day walnuts smoothie	Macronutrient-matched placebo smoothie	↑ PYY → Body weight → Waist circumference → Hip circumference → Waist/hip ratio → IMC → Fat body mass → Lean body mass → VAT mass
Lasa, 2014 [152]	High cardiovascular risk (PREDIMED study)	124	RCT, parallel-arm	1 year	Mediterranean diets supplemented with 30 g/day mixed nuts daily	Low-fat diet	*From baseline:* ↑ Adiponectin ↓ Adiponectin/leptin ratio ↓ Weight → BMI In women: ↓ Waist circumference In men: → Waist circumference
Wu, 2014 [153]	Healthy Caucasian men and postmenopausal women ≥ 50 years old	40	RCCT	8 weeks	43 g/day walnuts	Western-type diet	→ Adiponectin → Leptin

BMI: body mass index; RCT: randomized clinical trial; RCCT: randomized crossover clinical trial; T2DM: type 2 diabetes mellitus; BIA, bioelectrical impedance analysis; FFM, fat free mass; SCAT, (abdominal) subcutaneous adipose tissue; VAT, visceral adipose tissue; CT: total cholesterol; HDL-c: high density lipoprotein cholesterol; LDL-c: low density lipoprotein cholesterol; CAD: coronary artery disease; NCEP: National Cholesterol Education Program; PREDIMED: Prevención con Dieta Mediterránea; ↑: increase; →: maintenance or no effect; ↓: decrease.
